# Re-evaluation of *Streptococcus pneumoniae* carriage in Portuguese elderly by qPCR increases carriage estimates and unveils an expanded pool of serotypes

**DOI:** 10.1038/s41598-020-65399-x

**Published:** 2020-05-20

**Authors:** Sónia T. Almeida, Tânia Pedro, A. Cristina Paulo, Hermínia de Lencastre, Raquel Sá-Leão

**Affiliations:** 1 Laboratory of Molecular Microbiology of Human Pathogens, Instituto de Tecnologia Química e Biológica António Xavier, Universidade Nova de Lisboa, Oeiras, Portugal; 2 Laboratory of Molecular Genetics, Instituto de Tecnologia Química e Biológica António Xavier, Universidade Nova de Lisboa, Oeiras, Portugal; 3 Laboratory of Microbiology and Infectious Diseases, The Rockefeller University, New York, USA

**Keywords:** Microbiology, Bacteria, Infectious-disease epidemiology

## Abstract

*Streptococcus pneumoniae* (pneumococcus) is a leading cause of infections worldwide. Disease is preceded by asymptomatic colonization of the upper respiratory tract. Classical culture-based methods (CCBM) suggest that colonization in the elderly is <5%. Recently, use of qPCR has challenged these observations. We estimated pneumococcal carriage prevalence and serotypes among Portuguese elderly using qPCR and compared results with those obtained by CCBM. Nasopharyngeal and oropharyngeal paired samples (599 each) of individuals over 60 years living in nursing (n = 299) or family (n = 300) homes were screened for the presence of pneumococci by qPCR targeting *lytA* and *piaB*. Positive samples were molecular serotyped. Use of qPCR improved detection of pneumococci in oropharyngeal samples compared to CCBM: from 0.7% to 10.4% (p < 0.001) in the nursing home collection, and from 0.3% to 5.0% (p < 0.001) in the family home collection. No significant differences were observed between both methods in nasopharyngeal samples (5.4% *vs*. 5.4% in the nursing homes; and 4.3% *vs*. 4.7% in the family homes). Twenty-one serotypes/serogroups were detected by qPCR compared to 14 by CCBM. In conclusion, use of qPCR suggests that pneumococcal carriage in Portuguese elderly is approximately 10%, and unveiled a large pool of serotypes. These results are important to understand progression to disease and impact of pneumococcal vaccines in the elderly.

## Introduction

*Streptococcus pneumoniae* (or pneumococcus) is a leading cause of infectious diseases worldwide, such as otitis media, pneumonia, bacteremia and meningitis. The incidence of pneumococcal infections is highest at the extremes of age affecting disproportionally young children and the elderly^1–3^. This latter group is increasingly important in our society as the world population is aging, resulting in an increasing demand for strategies to maintain quality-adjusted life years in advanced ages^2^.

To prevent pneumococcal disease three vaccines are currently available and several are under investigation^4^. These vaccines target 10, 13, or 23 serotypes of the over 95 described to date^5^. The 13-valent pneumococcal conjugate vaccine (PCV13) is approved for all age groups and the 23-valent pneumococcal polysaccharide vaccine (PPV23) is approved for individuals over 2 years of age. Several countries have issued recommendations for vaccination of adults over 65 years of age with one or both of these vaccines^1,6,7^.

In Portugal, PCVs for children were commercially available through the private market until mid-2015 (PCV7 became available in June 2001, PCV10 in April 2009, and PCV13 in January 2010). In August 2015, PCV13 was introduced in the National Immunization Plan for all children born after January 2015^8^. PPV23 and PCV13 are commercially available for adults but its usage has been low (<10% by 2015)^9,10^.

Pneumococcal disease is always preceded by colonization of the upper respiratory tract, a phenomenon that is mostly asymptomatic^11^. Several studies have described high pneumococcal colonization rates (frequently over 60%) in young children^12,13^. By contrast, studies conducted in elderly populations have suggested, until recently, that pneumococcal colonization occurred at a very low prevalence (1–5%)^14–16^. These studies relied on the use of classical culture-based approaches in which swabs of the nasopharynx and/or oropharynx were plated in selective media and pneumococcal presumptive colonies were sub-cultured for species identification^17^. This strategy, recommended by the WHO, has a good specificity but a low sensitivity^17,18^.

Recently, new strategies to detect pneumococcal carriage based on real-time PCR (qPCR) have been proposed, validated, and are now being increasingly used^19–23^. The use of qPCR enables high sensitivity in samples where pneumococci are at low density. With such approaches, carriage prevalence in the elderly has been re-evaluated with studies suggesting it may range between 0–20% in healthy individuals and reach approximately 50% period-prevalence (within 7–9 weeks) in individuals with influenza-like illness^20–22,24^.

In Portugal, the prevalence of pneumococcal carriage among the elderly has been studied by classical culture-based methods only. A previous study conducted by our group estimated a carriage prevalence of 2.3%^15^.

The aim of this study was to re-evaluate the prevalence of nasopharyngeal and oropharyngeal colonization by *S. pneumoniae* in adults over 60 years of age using qPCR and compare the results with those obtained by classical culture-based approaches. We further characterized positive samples by serotyping and compared serotype distribution and diversity.

## Results

### Pneumococcal carriage

We evaluated pneumococcal carriage by real-time PCR targeting *lytA* and *piaB* genes in nasopharyngeal and oropharyngeal samples from 599 adults older than 60 years of age living in nursing homes (n = 299) and family homes (n = 300). Socio-demographic characteristics of the population were previously described^15^ and are summarized in Supplementary Table S1. In general, individuals living in a nursing home, were older, had less years of formal education, and were less active than those living in family homes. Results were compared to those obtained by classical culture-based methods.

**Figure 1:**
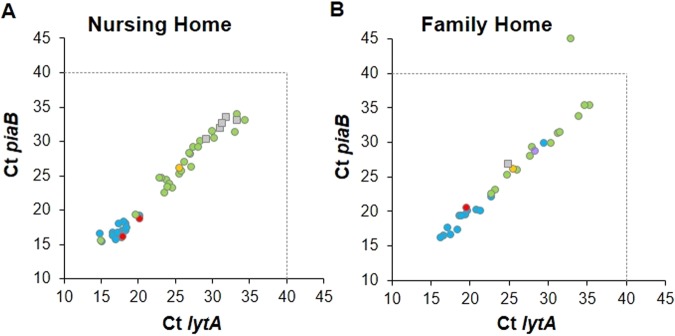
Detection of *S. pneumoniae* by real-time PCR in adults over 60 years of age living in nursing homes (**A**) and family homes (**B**). Blue circles, nasopharyngeal samples positive by culture; purple circles, nasopharyngeal samples negative by culture; red circles, oropharyngeal samples positive by culture; green circles, oropharyngeal samples negative by culture; yellow circles, positive control (*S. pneumoniae* TIGR4); grey squares, positive oropharyngeal samples in which the serotype was not determined. Dashed lines indicate the Ct value above which results were considered to be negative.

Ct values for *lytA* and *piaB* genes were not significantly different between samples from adults living in nursing homes compared to those living in family homes (adjusted GLM, p = 0.065 for *lytA*; p = 0.048 for *piaB*). However, in both groups, the geometric Ct mean was significantly lower in positive nasopharyngeal samples than in positive oropharyngeal samples (p < 0.001 for both *lytA* and *piaB*), suggesting higher quantity of pneumococci in the harvests of cultures originating from nasopharyngeal samples compared to those originating from oropharyngeal samples (Fig. 1 and Supplementary Table S2).

**Figure 2:**
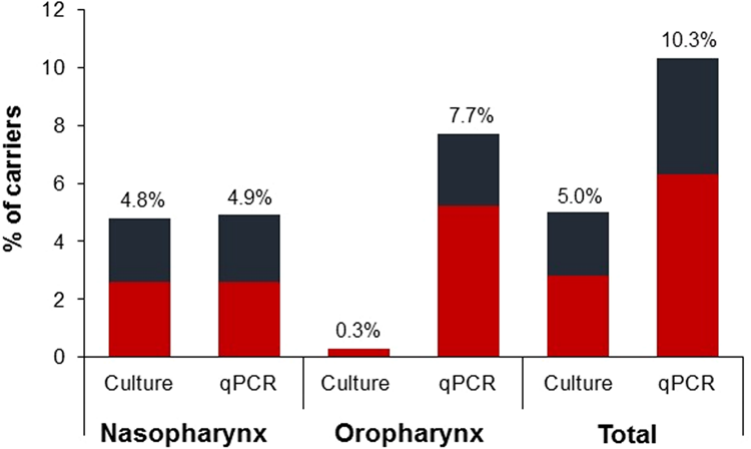
Proportion of pneumococcal carriers detected by classical culture-based methods or real-time PCR. Red bars, adults over 60 years of age living in nursing homes; blue bars, adults over 60 years of age living in family homes.

The use of real-time PCR, when compared with classical culture-based methods, increased significantly the detection of pneumococcal carriage in oropharyngeal samples from 0.7% to 10.4% (p < 0.001) in the nursing home collection, and from 0.3% to 5.0% (p < 0.001) in the family home collection. By contrast, no significant differences were observed in carriage detection in nasopharyngeal samples when both methods were compared: 5.4% vs. 5.4% in the nursing home collection; and 4.3% vs. 4.7% (p = 1.0) in the family home collection (Table 1 and Fig. 2).

**Table 1:** Detection of *S. pneumoniae* carriers according to sampling site and methodology used.

**Collection**	Participants n	Sampling Site n (%)								Carriers n (%)			
		Oropharynx (OP)				Nasopharynx (NP)							
		Culture positive	qPCR positive	Culture and/or qPCR positive	p-value	Culture positive	qPCR positive	Culture and/or qPCR positive	p-value	Culture positive (NP and/or OP)	qPCR positive (NP and/or OP)	Total	p-value
Nursing home	299	2 (0.7%)	31 (10.4%)	31 (10.4%)	**<0.001**	16 (5.4%)	16 (5.4%)	16 (5.4%)	NA	17 (5.7%)	38 (12.7%)	38 (12.7%)	**<0.001**
Family home	300	1 (0.3%)	15 (5.0%)	15 (5.0%)	<**0.001**	13 (4.3%)	14 (4.7%)	14 (4.7%)	1.0	13 (4.3%)	24 (8.0%)	24 (8.0%)	**0.003**

p-values determined using McNemar’s Chi-squared test for paired individuals.

Overall, in adults living in nursing homes*, S. pneumoniae* was more frequently detected in oropharyngeal samples than in nasopharyngeal samples: 10.4% vs. 5.4% (p = 0.0093), respectively; in adults living in family homes no significant differences were observed between the two sampling sites: 4.7% vs. 5.0% (p = 1.0) (Table 1). When results of the two sampling sites were combined, the use of real-time PCR, compared to classical culture-based methods, increased significantly the detection of *S. pneumoniae* carriers from 5.7% to 12.7% (p < 0.001) in the nursing home collection and from 4.3% to 8.0% (p = 0.0026) in the family home collection (Table 1).

### Molecular serotyping

Molecular serotyping allowed assignment of a serotype/serogroup in 70 (40 from oropharynx and 30 from nasopharynx) of the 76 qPCR positive samples. A single capsular type was detected in 67 qPCR pneumococcal positive samples and two serotypes were detected in three samples: one individual was co-colonized with serotypes 19A and 22A/F which were detected both in the nasopharynx and the oropharynx; other individual was co-colonized with serotypes 3 and 23A in the oropharynx. The six samples for which a serotype could not be assigned were all from the oropharynx (Fig. 1). Failure of assigning a capsular type to these samples suggests that they may have not been targeted by the primer set tested.

In total, twenty-one different serotypes/serogroups were detected among 76 samples that were positive for pneumococci by qPCR compared to 14 serotypes present in the 32 samples that were positive for pneumococci by classical culture-based methods. Serotype diversity (GSID = 0.897, 95% CI: 0.896–0.898) was slightly higher among samples detected by qPCR compared to those by classical culture-based methods (GSID = 0.879, 95% CI: 0.877–0.881). Serotypes/serogroups 15 A/F, 24 and 38, which are not included in any pneumococcal multi-valent vaccine, and serotypes/serogroups 7A/F, 10A, 12A/B/F and 20 potentially targeted by PPV23 (7F is also targeted by PCV13), were only detected in qPCR positive samples (Table 2).

**Table 2:** *S. pneumoniae* serotypes/serogroups detected in samples obtained from adults over 60 years old.

**Serotype/serogroup**	**Vaccine**^**a**^	**Number of samples with serotype/serogroup**			
		**Nursing homes**		**Family home**	
		**Classical culture-based approaches**^**b**^	**qPCR**^**c**^	**Classical culture-based approaches**^**b**^	**qPCR**^c^
3	PCV13/PPV23	—	—	2	3
5	PCV13/PPV23	—	1^d^	—	—
6A	PCV13/PPV23	—	—	2	2
6C/D	—	—	2	2 (6C)	4
7A/F	PCV13/PPV23	—	—	—	1
7B/C	—	—	—	1	1
9L	—	—	—	1	1
10A	PPV23	—	—	—	1
11A/D	PPV23	—	3	1 (11A)	1
12A/B/F	PPV23	—	—	—	1
15A/F	—	—	—	—	1
16F	—	—	—	1	2
17F	PPV23	1	3	—	—
19A	PCV13/PPV23	7	16	—	1
20	PPV23	—	2	—	—
22A/F	PPV23	2 (22F)	4	—	1
23A	—	4	6	2	3
23B	—	1	1	1	1
24	—	—	1	—	2
31	—	—	—	1	1
35F	—	3	6	1	1
38	—	—	—	—	1
ND	—	—	5	—	1

^a^Indicates whether the serotype/serogroup is potentially targeted by the 13-valent pneumococcal conjugate vaccine (PCV13: 1, 3, 4, 5, 6A, 6B, 7F, 9V, 14, 18C, 19A, 19F and 23F) or by the 23-valent polysaccharide vaccine (PPV23: 1, 2, 3, 4, 5, 6B, 7F, 8, 9N, 9V, 10A, 11A, 12F, 14, 15B, 17F, 18C, 19A, 19F, 20, 22F, 23F and 33F).

^b^Detection of pneumococci by identification and characterization of presumptive colonies grown on gentamicin blood agar, as described in the Methods section.

^c^Detection of pneumococci using a qPCR targeting *lytA* and *piaB* genes, as described in the Methods section.

^d^Given that this specific assay is known to yield false positive results, it was not taken into consideration.

ND, not determined.

Combined nasopharyngeal and oropharyngeal sampling did not yield significantly more different serotypes than each sampling site alone (14 serotypes/serogroups detected in nasopharyngeal samples and 17 detected in the oropharyngeal samples, p = 0.166 and p = 0.564, respectively).

The most frequent serotypes/serogroups were 19A (n = 17), 23A (n = 9), 35F (n = 7), 6C/D (n = 6), 22A/F (n = 5) and 11A/D (n = 4), which, together, accounted for 68.6% of all serotyped samples (Table 2). Serotypes 7B/C (n = 1), 9L (n = 1), 23B (n = 2) and 31 (n = 1) were only found in nasopharyngeal samples, while serotypes 7A/F (n = 1), 10A (n = 1), 12A/B/F (n = 1), 15A/F (n = 1), 20 (n = 2), 24 (n = 3) and 38 (n = 1) were only found in oropharyngeal samples (Table 2).

Overall, among the 62 carriers (38 living in nursing homes and 24 living in family homes) a maximum of 25.8% and 46.8% had serotypes potentially covered by PCV13, and PPV23, respectively.

**Figure 3:**
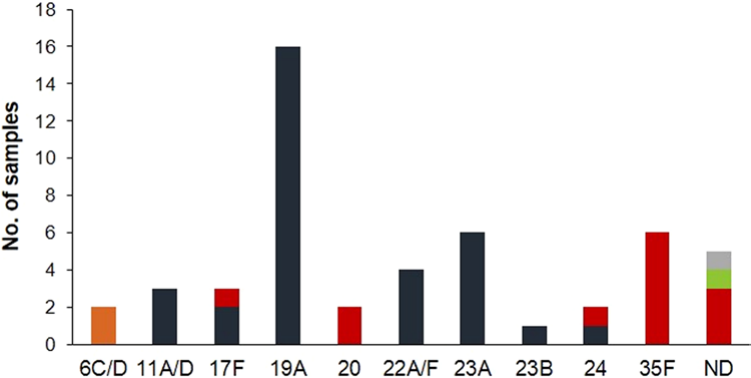
Serotype distribution of pneumococcal isolates carried by adults over 60 years of age living in nursing homes. Different colors indicate different nursing homes.

Of note, within nursing homes, there was evidence of cross-transmission with a common serotype being frequently isolated from multiple individuals (Fig. 3). This contributed to a lower serotype diversity in the nursing home collection (GSID = 0.778, 95% CI: 0.775–0.782) when compared to the family home collection (GSID = 0.919, 95% CI: 0.918–0.920).

## Discussion

In this study qPCR targeting *lytA* and *piaB* was used to investigate the prevalence of nasopharyngeal and oropharyngeal pneumococcal carriage in two collections of samples obtained from adults over 60 years of age living in either nursing homes or family homes. Positive samples were further characterized by molecular serotyping. These collections were previously characterized by classical culture-based methods as part of a much larger pneumococcal carriage study^15^.

In our previous study, as well as in studies from countries such as Belgium, Finland and Israel – which also used conventional culture followed by identification of presumptive colonies *S. pneumoniae* – pneumococcal carriage rates were estimated to be in the range of 3–5% among the elderly^14–16,25^.

In this study, use of qPCR increased the detection of pneumococci in oropharyngeal samples by approximately 15 times in both the nursing home and family home collections. By contrast, there was no significant added value in using qPCR to detect pneumococci in nasopharyngeal samples. The use of qPCR is known to be particularly valuable to detect pneumococci in samples that are highly polymicrobial and/or in which pneumococci are present at low density. In oropharyngeal samples both conditions tend to be common^19,20,22^. The nasopharynx, by contrast, is known to be less polymicrobial and pneumococci, when present, tend to thrive^15,26–29^.

When all results were compared, pneumococci were more frequently detected in oropharyngeal samples than in nasopharyngeal samples (7.7% vs. 5.0%, McNemar’s, p < 0.05) and there were also twice more individuals positive for pneumococci exclusively in oropharyngeal samples (n = 32) compared to individuals positive exclusively in nasopharyngeal samples (n = 16). These observations suggest that pneumococci, in the elderly, tend to preferentially colonize the oropharynx.

They also support the recommendation that to detect pneumococci in senior adults it is important to combine both nasopharyngeal and oropharyngeal samples and that qPCR (targeting more than one validated gene, such as *lytA* and *piaB*) should be used when analyzing oropharyngeal samples^19,20,22^. In our study, half of the pneumococcal carriers were only detected in these conditions.

Overall, we estimated that approximately 10% of the individuals were pneumococcal carriers independently of whether they lived in a nursing home or in a family home. These estimates are lower than those described in studies conducted in Italy or the Netherlands, which were closer to 20%^22,24^. These differences are likely to be due to seasonality: the latter studies were carried out in winter or winter/spring while ours took place throughout the year. In fact, a variation between 0–17% in pneumococcal carriage prevalence among adults over 65 years of age was noted in the US longitudinal study of Branche *et al*.^21^.

While we did not observe differences in density of colonization between individuals living in nursing homes or family homes, we observed (unsurprisingly) lower serotype diversity and evidence for cross-transmission within nursing homes. These are settings where individuals are often confined and where transmission of infectious agents is often facilitated^30^.

Molecular serotyping of qPCR pneumococcal positive samples enabled assignment of a serotype/serogroup to most (92%) samples. Furthermore, the number of different serotypes that were detected increased by 50% (21 in qPCR samples vs. 14 by classical culture-based processed samples) expanding considerably the pool of serotypes detected in the population. This included non-vaccine serotypes and vaccine serotypes that, otherwise, would remain undetected and that may be important to monitor intervention strategies such as the use of pneumococcal vaccines.

In conclusion, carriage of pneumococci in senior adults is significantly higher than the one estimated by classical culture-based methods alone. Our current estimates suggest that, in Portugal, it is approximately 10%. The high number of serotypes circulating in this population and the transmission observed within nursing homes warrant additional studies aimed to assess its implications in disease. As the number and proportion of aged individuals increases in societies worldwide, accurate estimates of pneumococcal carriage prevalence and circulating serotypes in this group are crucial to better understand colonization dynamics, serotype disease potential, and impact of pneumococcal vaccines in this age group.

## Methods

### Study participants and samples

This study was nested on a study previously described^15^. Briefly, pneumococcal carriage prevalence and associated risk factors were studied among 3,361 adults older than 60 years of age, between April 2010 and December of 2012. For each participant, one nasopharyngeal and one oropharyngeal sample were obtained. Pneumococci were isolated and identified by routine procedures based on culture-based methods^31,32^, and serotyped by conventional multiplex PCR and/or by the Quellung reaction^33,34^. Briefly, in the initial study, swabs were plated on gentamycin blood agar and incubated overnight at 37 °C in anaerobiose jars. On the following day, suspected pneumococcal colonies were isolated and identified by routine culture methods. All the remaining total bacterial growth was collected and frozen at −80 °C.

In the current study, two subsets of samples of the initial study were re-examined. One set corresponded to 600 paired samples (300 nasopharyngeal and 300 oropharyngeal obtained from 300 individuals) randomly selected from a pool previously obtained from 3,062 individuals living in family homes. The second set corresponded to 598 samples (299 nasopharyngeal and 299 oropharyngeal) obtained from all individuals living in nursing homes at the time of the study^15^.

### Identification and serotyping of *S. pneumoniae* by molecular methods

Total bacterial growth was thawed on ice, vortexed for 20 seconds and 200 µl were transferred into a sample tube with 200 µl of a lysis buffer (MagNA Pure Compact Nucleic Acid Isolation Kit, Roche Diagnostics GmbH), and incubated for 20 min at 37 °C. Total DNA was then extracted using the MagNA Pure Compact instrument (Roche Diagnostics GmbH) as recommended by the manufacturer.

Pneumococcal carriage was evaluated by real-time PCR, targeting two pneumococcal genes: *lytA* (major pneumococcal autolysin) and *piaB* (iron uptake ABC transporter lipoprotein PiaB)^19,35^. Reactions for both genes were performed in a final volume of 25 µl, containing 1x FastStart TaqMan Probe Master (Roche), 150 nM of each primer, 75 nM of probe and 2.5 µl of total DNA. DNA amplification was performed in the CFX96 Real-Time System Amplification (Bio-Rad) using the following conditions: 95 °C for 10 min, followed by 45 cycles of 95 °C for 15 sec and 60 °C for 1 min. Samples were considered positive for pneumococci when both genes had cycle threshold (Ct) values below 40.

*S. pneumoniae* capsular types were accessed in positive samples by uniplex qPCR following a pooling strategy as previously described^36^. Pools of five samples containing 2 µl of each sample were tested. Each pool was used as template for qPCR in a total volume of 25 µl. If a Ct<40 was obtained in a given pool of samples, these were tested individually for the serotype that originated the signal. In this latter case, 2.5 µl of DNA was used per reaction. Samples were considered positive for a given serotype when Ct values were below 40. A panel of 23 sets of primers and probes was used targeting serotypes or serogroups: 1, 2, 3, 4, 5, 6A/6B/6C/6D, 7A/F, 9A/V, 11A/D, 12A/12B/12F/44/46, 14, 15A/F, 16F, 18A/18B/18C/18F, 19A, 19F, 22A/F, 23A, 23F, 33A/33F/37^37^, 8, 10A/B and 38^38^. Samples were considered positive for a given serotype when Ct values were below 40. Following previous studies that indicated that non-pneumococcal streptococci in the oropharynx can confound molecular serotyping assays, we disregarded positive results obtained by qPCR for serotypes 4 and 5^20,26,39^. In addition, conventional multiplex PCR was used to detect serotypes 7B/7C/40, 9N/L, 15B/C, 17F, 20, 21, 23B, 24, 31, 34, 35B, 35F/47F and non-typeables using primers previously described^33,40^.

### Statistical analysis

The geometric mean was used to summarize the distribution of Ct values from *lytA* and *piaB* of nasopharyngeal and oropharyngeal samples. To look for associations between Ct values from nursing home *vs* family home and nasopharynx vs oropharynx an adjusted generalized linear model (GLM) using a Gaussian distribution and a log link function was used.

The McNemar’s test was used to compare culture and real-time PCR methods based on paired individuals. The Chi-square test was used to compare prevalence of *S. pneumoniae* between nasopharyngeal and oropharyngeal samples and between nursing home and family home by real-time PCR or culture-based methods. A p-value of <0.05 was considered statistically significant for all the tests used. The Gini-Simpson index of diversity (GSID) was used to calculate serotype diversity. All analyses were performed using R version 3.2.3^41^.

### Ethics statement

This study was conducted in accordance with the European Statements for Good Clinical Practice and the declaration of Helsinki of the World Health Medical Association. In addition, it was registered and approved at health care centers of Oeiras and Montemor-o-Novo that report to Administração Regional de Saúde (ARS, “Regional Health Administration”) of Lisboa e Vale do Tejo, and Alentejo, respectively, from the Ministry of Health. Informed written consent was obtained from all participants. All samples and questionnaires were attributed a numeric code and were processed anonymously.

## Supplementary information

Supplementary information.
