# Supplementary material for: Re-evaluation of Streptococcus pneumoniae carriage in Portuguese elderly by qPCR increases carriage estimates and unveils an expanded pool of serotypes
Source: Sci Rep. 2020 May 20;10:8373. doi: 10.1038/s41598-020-65399-x (PMC7239868; doi:10.1038/s41598-020-65399-x)
Supplement: Supplementary file 1 — Supplementary information. [file 41598_2020_65399_MOESM1_ESM.docx]

**Re-evaluation of *Streptococcus pneumoniae* carriage in Portuguese elderly by qPCR increases carriage estimates and unveils an expanded pool of serotypes**

Sónia T. Almeida^a^, Tânia Pedro^a^, A. Cristina Paulo^a^, Hermínia de Lencastre^b,c^ and Raquel Sá-Leão^a^*

^a^Laboratory of Molecular Microbiology of Human Pathogens, Instituto de Tecnologia Química e Biológica António Xavier, Universidade Nova de Lisboa, Oeiras, Portugal; ^b^Laboratory of Molecular Genetics, Instituto de Tecnologia Química e Biológica António Xavier, Universidade Nova de Lisboa, Oeiras, Portugal; ^c^Laboratory of Microbiology and Infectious Diseases, The Rockefeller University, New York, USA

**Supplementary Table S1.** Socio-demographic characteristics of the population.

| Variable | Participants^1^ (total=3361) | Nursing home collection^2^  (n=299) | Family home collection^2^  (n=300) |
| --- | --- | --- | --- |
| Living area |  |  |  |
| urban | 1945 (57.9%) | 27 (9.3%) | 101 (33.7%) |
| rural | 1416 (42.1%) | 272 (90.7%) | 199 (66.3%) |
| Mean age (yrs) | 74.5 ± 8.2 | 82.5 ± 6.9 | 73.8 ± 7.7 |
| Gender |  |  |  |
| female | 1935 (57.6%) | 194 (64.9%) | 170 (56.7%) |
| male | 1426 (42.4%) | 105 (35.1%) | 130 (43.3%) |
| Years of school education |  |  |  |
| 0 | 315 (9.4%) | 72 (24.1%) | 33 (11.0%) |
| 1-4 | 2799 (83.3%) | 226 (75.6%) | 254 (84.7%) |
| ≥ 5 | 246 (7.3%) | 1 (0.3%) | 13 (4.3%) |
| Retirees |  |  |  |
| retired | 3015 (89.7%) | 299 (100%) | 266 (88.7%) |
| active | 346 (10.3%) | 0 (0.0%) | 34 (11.3%) |
| Housing |  |  |  |
| family home | 3062 (91.1%) | 0 (0.0%) | 300 (100%) |
| retirement home | 299 (8.9%) | 299 (9.3%) | 0 (0.0%) |
| Weekly contact with children ≤ 6 yrs |  |  |  |
| yes | 650 (19.3%) | 1 (0.3%) | 46 (15.3%) |
| no | 2711 (80.7%) | 298 (99.7%) | 254 (84.7%) |
| Recreational activities |  |  |  |
| at least one activity | 1119 (33.3%) | 9 (3.0%) | 131 (43.7%) |
| club | 339 (10.1%) | 4 (1.3%) | 30 (10.0%) |
| day center | 652 (19.4%) | 6 (2.0%) | 94 (31.3%) |
| senior university | 51 (1.5%) | 0 (0.0%) | 1 (0.3%) |
| other | 99 (2.9%) | 0 (0.0%) | 10 (3.3%) |
| Smoker |  |  |  |
| yes | 126 (3.7%) | 5 (1.7%) | 12 (4.0%) |
| no | 3235 (96.3%) | 294 (98.3%) | 288 (96.0%) |
| Vaccination with PPV23 |  |  |  |
| yes | 122 (3.6%) | 8 (2.7%) | 11 (3.7%) |
| no | 3239 (96.4%) | 291 (97.3%) | 289 (96.3%) |

^1^Data described in Almeida *et al*.^15^.

^2^Data relative to samples analyzed in this study.

**Supplementary table S2.** Geometric mean of the distribution of Ct values for *lytA* and *piaB* genes of oropharyngeal and nasopharyngeal samples.

|  | Geometric mean ± Geometric Sd | |
| --- | --- | --- |
|  | *lytA* | *piaB* |
| Oropharyngeal samples | 26.79 ± 1.19 | 26.90 ± 1.29 |
| Nasopharyngeal samples | 19.11 ± 1.17 | 18.52 ± 1.19 |
